# The Combination of Shear Wave Elastography and Platelet Counts Can Effectively Predict High-Risk Varices in Patients with Hepatitis B-Related Cirrhosis

**DOI:** 10.1155/2021/6635963

**Published:** 2021-04-07

**Authors:** Xiaoyu Xie, Yuemin Feng, Zhuozhen Lyu, Le Wang, Yao Yang, Yuping Bai, Chenxi Liu, Hao Wu, Wanhua Ren, Qiang Zhu

**Affiliations:** ^1^Department of Gastroenterology, Shandong Provincial Hospital, Cheeloo College of Medicine, Shandong University, Jinan, Shandong, China; ^2^Department of Infectious Disease, Shandong Provincial Hospital, Cheeloo College of Medicine, Shandong University, Jinan, Shandong, China; ^3^Department of Geriatrics, Gastroenterology, Shandong Provincial Hospital, Cheeloo College of Medicine, Shandong University, Jinan, Shandong, China; ^4^Department of Gastroenterology, The First Affiliated Hospital of Xinjiang Medical University, Urumqi, 830054, China

## Abstract

**Background:**

Baveno VI criteria, based on liver stiffness (LS) measured by transient elastography and platelet counts (PLT), have been proposed to avoid unnecessary endoscopy screening for high-risk varices (HRVs). However, the cut-off value of LS measured by 2D-SWE and PLT to predict HRVs in compensated hepatitis B-related cirrhotic patients remains unknown.

**Aims:**

To prospectively analyze the cut-off of the combination of LS measured by 2D-SWE and PLT in predicting HRVs and the influence of antiviral therapies in its efficacy.

**Methods:**

Serum parameters, LS, and endoscopy results were obtained from 160 compensated hepatitis B-related cirrhotic patients. The accuracy of the combined algorithm was assessed in the whole cohort and subgroups with or without consecutive antiviral therapies in the past 6 months.

**Results:**

In the whole cohort, the optimal cut-off value of LS for HRVs was 14.5 kPa. Patients with a LS value < 14.5 kPa with a PLT value > 110 × 10^9^/L can be excluded from HRVs (NPV = 0.99, endoscopy saved rates = 0.68). Conversely, a LS value of ≥14.5 kPa and a PLT value of ≤110 × 10^9^/L indicated HRVs, with accurate rates of 82.35%, and 10.63% of patients can avoid additional endoscopy screening. Moreover, antiviral therapy had no significant effect on the accuracy and rates saved from further endoscopy screening, when comparing patients with or without antiviral therapies (all *p* values > 0.05).

**Conclusions:**

The combination of LS (14.5 kPa) measured by 2D-SWE and PLT (110 × 10^9^/L) can predict HRVs accurately in compensated hepatitis B-related cirrhotic patients without significant interference of antiviral therapy histories.

## 1. Introduction

Hepatic cirrhosis and portal hypertension are the common consequences of chronic hepatitis B infection. Patients with clinically significant portal hypertension are at risk of developing esophageal varices (EVs), and varix bleeding is one of the major causes of death in patients with hepatitis B-related cirrhosis [1]. Due to the high rates of bleeding and mortality, screening EVs at the time of cirrhosis diagnosis is generally recommended [2]. The patients detected with high-risk varices (HRVs) need to receive the primary prophylaxis to prevent variceal bleeding and reduce the risk of decompensation and death. Upper gastrointestinal endoscopy is the universally accepted gold standard for the diagnosis of HRVs, and measurement of the hepatic venous pressure gradient (HVPG) is currently the best available method to evaluate the presence and severity of portal hypertension. However, patients are not readily accepted because of their invasiveness and higher costs; thus, there is still an urgent need for developing straightforward and noninvasive markers for HRVs.

Several noninvasive indexes have been tested to predict HRVs in cirrhotic patients, such as Doppler ultrasonography, computed tomography (CT), magnetic resonance imaging (MRI), and elastography techniques. CT and MRI allow direct visualization of esophageal varices after intravenous contrast administration, whereas the cost of both methods is higher compared to other noninvasive methods. Various indexes measured by Doppler ultrasonography have been proposed as noninvasive predictors for EVs. However, all these parameters fail to accurately predict the presence of large varices and display a lower accuracy than that of liver elastography [3]. Transient elastography (TE) is a one-dimensional ultrasound-based technology, and some studies have shown that TE displays an excellent diagnostic accuracy for predicting the presence of HRVs [4]. The opposite view indicates that TE is not suitable for patients with ascites and is performed without direct B-mode imaging guidance, which will impede the identification of the appropriate region for assessment [5, 6]. Recently, two-dimensional shear wave elastography (2D-SWE) has been merged into traditional ultrasound machines, thus, enables the operator to analyze a broader region of interest in real-time, further improving the applicability of elastography [7]. Current results from the meta-analysis display a better accuracy for the hepatic fibrosis estimation by 2D-SWE as compared with TE [8]. Notably, due to the different technical principles, LS values measured by 2D-SWE are different from values by TE [9, 10]. Thus, the cut-off acquired from TE for predicting HRVs is not applicable for 2D-SWE. Moreover, cirrhosis etiology also has a substantial impact [11], therefore, the cut-off of LS measured with 2D-SWE for the diagnosis of HRVs still needs to be determined in patients with hepatitis B-related cirrhosis.

Serum indexes are another kind of noninvasive markers for the prediction of HRVs. Platelet counts (PLT) have been attracted more attention among these indexes, since thrombocytopenia is mostly associated with portal hypertension in cirrhotic patients. Another advantage of PLT lies in its easy availability in the clinical practice without an increase in expense for cirrhotic patients [12]. Moreover, combining PLT with other indexes can further improve the accuracy for the estimation of HRVs [13], thus, Baveno VI and its expanded criteria, based on liver stiffness (LS) measured by transient elastography and PLT, have been proposed to avoid unnecessary endoscopy screening for HRVs [14, 15]. As mentioned above, the LS value measured by 2D-SWE is different from that by TE, and hepatitis B-related cirrhosis is also different from other etiologies. Therefore, the cut-off value of LS detected by 2D-SWE and PLT to predict HRVs remains unknown.

In this prospective study, we recruited 160 compensated hepatitis B-related cirrhotic patients who were ready to undergo 2D-SWE and endoscopic examinations. We aimed to investigate the cut-off and diagnostic efficacy of the combination of the LS measured by 2D-SWE and PLT to predict HRVs.

## 2. Materials and Methods

### 2.1. Patients

Patients with compensated hepatitis B-related cirrhosis at Shandong Provincial Hospital, Cheeloo College of Medicine, Shandong University, were recruited in this prospective study from May 2017 to June 2018. The inclusion criteria were (1) presence of hepatitis B surface antigen for at least six months and (2) cirrhosis detected by liver histology or unequivocal clinical and imaging data. The exclusion criteria were (1) coinfection with hepatitis C virus or other chronic liver diseases; (2) history of liver surgery and liver transplantation; (3) portal or hepatic vein thrombosis detected by imaging techniques; (4) previous treatments with transjugular intrahepatic portosystemic shunts, EVs banding ligations, or nonselective beta-blockers; (5) moderate-to-severe ascites; (6) hepatic encephalopathy; (7) the history or presence of variceal hemorrhage; (8) AST and/or ALT elevation > 5 × the upper limit of normal; (9) right heart failure; and (10) obstructive jaundice. Informed consent was obtained from all participants in this study. All procedures followed the 1964 Declaration of Helsinki and were approved by the Ethics Committee of Shandong Provincial Hospital. This study has been registered in the Chinese Clinical Trial Registry (1900020560).

### 2.2. Liver Stiffness Assessed by Two Dimensional-Shear Wave Elastography (2D-SWE)

Two experienced technicians (both of them have conducted more than 500 examinations), blinded to the upper gastrointestinal endoscopy results, performed 2D-SWE (Supersonic Imagine, Aix-en-Provence, France) with a convex broadband SC6-1 probe. After an overnight fast, 2D-SWE measurements were performed within 1 week after admission. Readings were taken in three different regions of interest (ROI) in the right lobe of the liver. The measurement of LS should be performed at least 10 mm below the liver capsule. The final LS value was calculated as the mean of the recorded acquisitions. Each acquisition was considered successful if the following three conditions are met (more than two-thirds of the elastography signal filled the region of interest (ROI), standard deviation (SD) of the LS value was ≤1.75 kilopascals (kPa), and big vessels and biliary tracts were not included in ROI). Measurements not fulfilling these requirements were considered failed, and such patients were excluded from the study.

### 2.3. Endoscopic Evaluation

EVs were detected by one expert endoscopist (having conducted more than 200 examinations per year) to avoid the influence of interobserver variability. Endoscopies were performed within 1 week after admission. EVs were staged according to the previously published criteria as follows: grade I: varices were flattened by insufflation; grade II: varices nonconfluent and protruding in the lumen despite insufflation; grade III: confluent varices were not flattened by insufflation [14]. The presence of red signs was also recorded in all patients. HRVs were defined as EVs grade 1 with red signs or grade 2 or higher.

### 2.4. Other Clinical Data

The demographic data and blood samples of patients were obtained at the time of hospital admission and were performed at the same admission as endoscopic and hepatic elastography evaluation. Blood samples were collected after a 12 h, overnight fast to detect alanine aminotransferase (ALT), aspartate aminotransferase (AST), gamma-glutamyl transpeptidase (GGT), total bilirubin (TBIL), albumin (ALB), prothrombin time (PT), international normalized ratio (INR), platelet count (PLT), serum creatinine, and hepatitis B DNA load. Liver function was assessed by the Child-Pugh classification and model for end-stage liver disease (MELD) score [16, 17]. The history of antiviral therapy was defined as consecutive antiviral treatment with nucleos(t)ide analogues or interferons in the past 6 months.

### 2.5. Statistical Analyses

Statistical analyses were performed with STATA 14.0 (College Station, TX, USA) and MedCalc 11.2 (MedCalc, Mariakerke, Belgium). The Shapiro-Wilk test was used for testing the distribution of numerical variables. Continuous variables were presented as mean ± SD or median (interquartile range) and categorical variables as numbers and percentages. Intergroup differences were assessed for significance using the Chi-squared test, Mann–Whitney test, Student's *t*-test, or Kruskal-Wallis test. Missing data were interpolated using the Expectation-Maximization method [18]. Results were reported as odds ratios (ORs) and 95% confidence intervals (CIs) whenever appropriate.

To avoid the omission of meaningful factors, variables significant in univariate analysis (*p* < 0.1) were included in the multiple regression models. Relationships between these parameters were characterized using Spearman correlation coefficients. The performance of noninvasive tests was assessed in terms of sensitivity, specificity, positive predictive value (PPV), negative predictive value (NPV), likelihood ratio, false-positive rate (FPR), false-negative rate (FNR), and area under the receiver operating characteristic curves (AUC). A comparison between AUCs was made by the Hanley and McNeil method [19]. Optimal cut-off values were chosen to maximize the sum of sensitivity and specificity. The calibration of tests was summarized by the Hosmer-Lemeshow goodness-of-fit test and observed/expected calibration plots. Furthermore, risk nomograms were constructed from logistic regression with LS and PLT as continuous predictors. The performance of combined noninvasive tests for predicting or excluding HRVs was assessed by the number and percentage of spared endoscopies and diagnostic accuracy. All statistical tests were two-sided. A *p* value of < 0.05 denoted statistical significance.

## 3. Results

### 3.1. Characteristics of Included Patients

**Figure 1:**
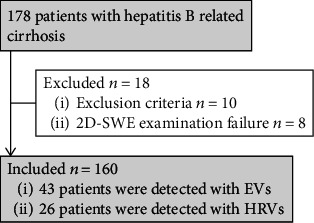
Flowchart shows patient selection in the study. Abbreviations: EVs: esophageal varices; HRVs: high-risk varices; SWE: shear wave elastography.

In this prospective study, 178 patients were initially recruited. Ten patients were excluded for the following reasons: coinfection with hepatitis C virus (*n* = 2), history of liver surgery (*n* = 2), portal or hepatic vein thrombosis detected by imaging techniques (*n* = 5), and previous treatments with EVs banding ligations (*n* = 1). Eight patients failed 2D-SWE examinations, resulting in 160 patients in the final analysis (Figure 1). The success rate was 95.24% (160/168). Analysis for failed examinations demonstrated that two patients had abdominal distension before the examination, and the other six patients were overweight (BMI: 29.68 ± 1.41 vs. 24.61 ± 0.27, *p* < 0.001). According to multivariate analysis, BMI was the only factor associated with unsuccessful LS measurements (OR 1.610, 95% CI: 1.205-2.150). In patients with successful measurements, the IQR/median was 0.051 ± 0.032 (range: 0.003-0.177), thus, all patients fulfilled the reliable criteria recommended by EFSUMB guidelines [20]. The concordance between the three values was almost perfect: ICC = 0.994, 95% CI: 0.993-0.996.

The prevalence of EVs was 26.88% (43/160), and HRVs were present in 26 (16.25%) patients. In addition, patients with antiviral therapy histories accounted for 36.88% (59/160) of the total cases, and the prevalence of HRVs in patients with antiviral histories was 20.34% (12/59), which was not significantly different from patients without antiviral histories (*p* = 0.284). Clinical characteristics of the included patients are summarized in Table 1.

### 3.2. 2D-SWE Displayed Better Performance for excluding High-Risk Varices in Patients with Hepatitis B-Related Cirrhosis

**Figure 2:**
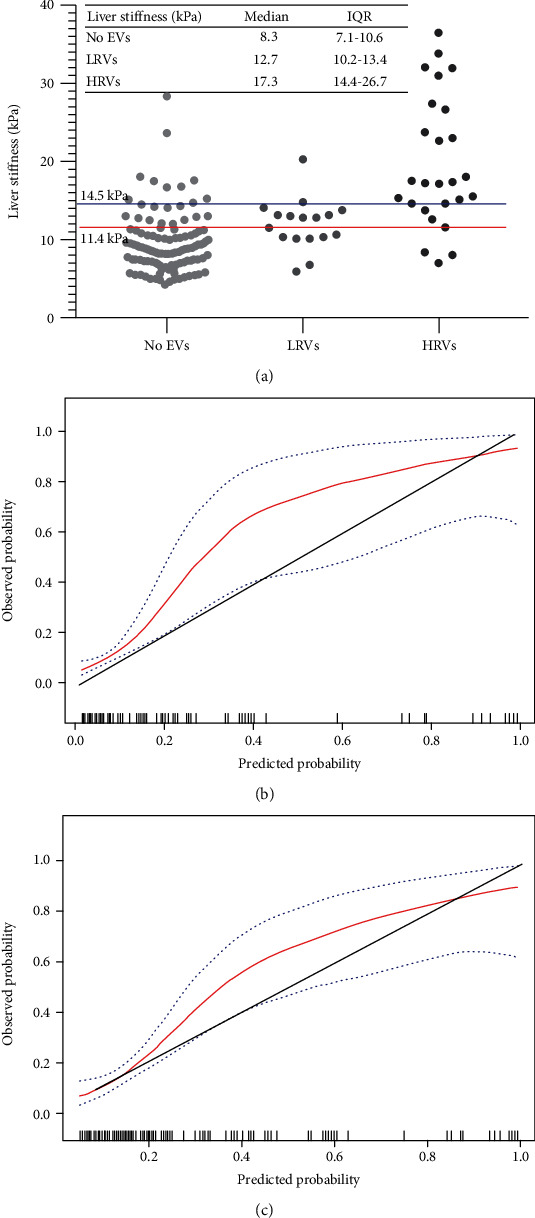
Distribution of liver stiffness was subdivided based on the absence of EVs, presence of LRVs or HRVs in all patients (a). The red and blue horizontal lines represent cut-offs of 2D-SWE for varices of all sizes and high-risk varices, respectively. Calibration plot of 2D-SWE (bootstrap resampling times = 500) for high-risk varices and varices of all sizes in all patients (b) and (c). Calibration slopes graph the agreement between predicted probability on the *x*-axis and observed proportion on the *y*-axis. The black dashed line represents perfect calibration, with 100% agreement. Red line represents the performance of 2D-SWE, and blue dashed line represents 95% confidence intervals of observed probabilities. Abbreviations: EVs: esophageal varices; LRVs: low-risk varices; HRVs: high-risk varices; 2D-SWE: two-dimensional shear wave elastography; kPa: kilopascals.

To assess diagnostic accuracy of 2D-SWE for EVs, LS values of no EVs, low-risk EVs, and HRVs were compared in the whole cohort (*p* < 0.001) (Figure 2(a)).  Table 2 was created as a summary of the entire cohort to assess the discrimination of 2D-SWE in diagnosing HRVs and varices of all sizes. Notably, LS exhibited good performance in excluding HRVs and all size varices with both NPVs ≥ 90%, whereas could not rule in HRVs and all size varices with adequate specificity and reliability due to the low PPVs (<65%). Using the optimal cut-off of 14.5 kPa for HRVs, the FPR and FNR of the examination were 9.70% (13/134) and 23.08% (6/26), respectively. A cut-off set at 11.4 kPa for all size varices had FPRs at 20.51% (24/117) and FNRs at 23.26% (10/43), respectively.

Moreover, the calibration of 2D-SWE was good by the Hosmer-Lemeshow test (*p* = 0.426), but slightly overestimated the probability of HRVs (Figure 2(b)). Similar results were obtained for all size varices shown in Figure 2(c) and tested by the Hosmer-Lemeshow method (*p* = 0.116).

### 3.3. The Combination of Liver Stiffness with Platelet Counts Improved the Accuracy of 2D-SWE for HRVs

To improve the accuracy of 2D-SWE, multivariable analysis for significant indicators in Table 1 established LS values; PLT, MELD score, and ALB concentration were independently associated with the presence of HRVs (Table S1). Based on the recommendation of Baveno VI guidelines and the diagnostic efficacy for HRVs (Table S2), the combination of LS and PLT was firstly investigated by a risk nomogram to determine the probability of HRVs (Figure S1). The combined algorithm displayed a higher AUC than LS alone (0.923, 95% CI 0.871-0.959 vs. 0.881, 95% CI 0.820-0.927, *p* = 0.157).

To further make a simple algorithm in the clinic, PLT's cut-off (110 × 10^9^/L) from the expanded Baveno VI criteria was verified [15]. Patients who meet the criteria (LS < 14.5 kPa and PLT > 110 × 10^9^/L) were less likely to be diagnosed with HRVs (NPV = 99.07%). One hundred and eight out of the 160 patients (67.50%) fulfilled the criteria and, consequently, avoided endoscopy screening. By combining LS (≥14.5 kPa) and PLT (≤110 × 10^9^/L) to assess HRVs, patients can only be diagnosed with 82.35% certainty, and 17 (10.63%) patients can avoid endoscopy screening. Overall, 125 patients (78.13%) could avoid endoscopic examinations, and the accuracy was 96.80% (121/125), which was better than that of LS alone (96.80% vs. 88%, *p* = 0.008) (Table 3).

### 3.4. Antiviral Therapy Histories Had No Significant Impact on the Combined Algorithm for Predicting High-Risk Varices

A subgroup analysis was conducted to investigate the impact of antiviral therapy histories on the performance of LS measured by 2D-SWE in the prediction of HRVs. The AUC was higher in patients without antiviral history than those who received the treatment (0.970, 95% CI 0.915-0.994 vs. 0.772, 95% CI 0.644-0.871, *p* = 0.016). Moreover, all patients who were underestimated using a cut-off of 14.5 kPa had antiviral therapy histories. Thus, patients with antiviral therapy histories had a higher FNR compared with those without antiviral histories (50% vs. 0%, *p* = 0.003), but the rates of discordance and FPRs were not statistically significant (15.25% vs. 9.90%, *p* = 0.313; 6.38% vs. 11.49%, *p* = 0.340). Furthermore, the logistic regression analysis showed that AST values were associated with underestimated results by 2D-SWE in patients with HRVs (OR = 0.891; 95% CI 0.795-0.997, *p* = 0.045). Overall, the above results indicated that the history of antiviral therapy was an important confounding factor that may underestimate HRVs by attenuating hepatic cirrhosis and inflammation.

On the other hand, the combined algorithm exhibited a significantly lower FNR than LS alone in patients with antiviral therapy histories (50.00% vs. 8.33%, *p* = 0.025), and only one patient was misclassified by the combined algorithm. Moreover, significantly lower discordance rates (9.90% vs. 0.99%, *p* < 0.001) and FPRs (11.49% vs. 1.15%, *p* < 0.001) were observed for the combined algorithm in patients without antiviral therapy histories.

**Figure 3:**
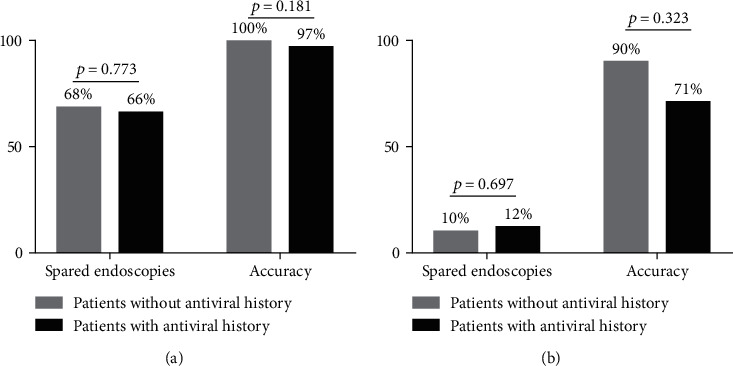
Performance of the combined algorithm in ruling out (a) and ruling in (b) high-risk varices in subgroups according to antiviral therapy histories.

The accuracy and endoscopy-spared rates of combined algorithms for excluding and predicting HRVs in patients with antiviral therapy histories were 97%, 66%, 71%, and 12%, respectively. No significant difference was observed for endoscopy-spared rates and diagnostic accuracy in a pairwise comparison between patients with and without antiviral therapy histories (all *p* > 0.05) (Figure 3).

## 4. Discussion

This study assessed the accuracy of combining the LS value, as measured by 2D-SWE, and the PLT value for predicting HRVs in patients with hepatitis B-related cirrhosis. Our findings demonstrated that in comparison with the LS value alone, this combined algorithm could further improve the accuracy of predicting HRVs. Nearly all patients without HRVs could be excluded and may be used as a first-line screening method to identify high-risk patients. Besides, the combination of LS with PLT displayed excellent accuracy even in patients with histories of antiviral treatment.

One of the major concerns for using 2D-SWE in cirrhotic patients is its feasibility and applicability. In this study, failed measurements only accounted for 4.76% (8/168). This desired result could be attributed to the technique's improvement by integrating 2D-SWE into traditional ultrasound machines, thereby ensuring the homogeneity of the elastic image [6]. Additionally, consistent with the study results by Procopet et al. [21], obesity and abdominal distensions were the leading cause of the failure in this technique. These factors will affect the depth of elastography quantification, which correlates with the accuracy and success rates of 2D-SWE directly. Moreover, the subcutaneous fat may also alter the pattern of the shear waves, leading to a lower rate of success [22]. It will be interesting to determine whether this technical problem can be resolved to further improve the applicability of 2D-SWE.

Another concern about predicting HRVs using 2D-SWE in compensated hepatitis B-related cirrhotic patients is its diagnostic efficacy. In the present study, LS showed higher NPVs than PPVs, which could probably be due to the lower prevalence of EVs. In this cohort, the prevalence of EVs and HRVs was 26.88% and 16.25%, respectively, and the differences in etiology may explain this lower prevalence [23]. This aspect can also explain the differences in cut-off values from other studies, and one typical example is that cut-off values of HRVs were higher for patients with alcoholic cirrhosis than those with viral cirrhosis [11]. One limitation of 2D-SWE lies in its low PPV for EVs, which is in the same line with results from TE [5]. We speculated that intrahepatic factors (i.e., increased vascular resistance caused by hepatic fibrosis) correlated well with the portal pressure in early stages, but the presence of EVs was also affected by extrahepatic factors [24]. Accordingly, it is imperative to combine LS with extrahepatic indices associated with portal hypertension (i.e., PLT) to improve the accuracy of 2D-SWE.

In order to further improve the diagnostic accuracy for ruling out HRVs, the expanded Baveno VI guidelines proposed that patients with compensated advanced chronic liver disease with an LS value by TE of <25 kPa and a platelet count >110 × 10^9^ cells/L can avoid the need for screening endoscopy [15]. However, due to the different technical principles, LS values measured with 2D-SWE were different from TE. On the other hand, Stefanescu et al. pointed out that LS values suggested by Baveno VI guidelines, but acquired from 2D-SWE, do not perform well for predicting the presence of any varices [25]. Therefore, more evidence is still required concerning the prediction of HRVs. We reported that the combination of LS (14.5 kPa) with PLT (110 × 10^9^/L) improved the ability to exclude patients with HRVs and reduced the need for endoscopy by 68%. Only one patient was misclassified, whose clinical characteristics were LS = 12.5 kPa, PLT = 225 × 10^9^/L, BMI = 29.38 kg/m^2^, implying the influence of obesity on LS measurement or instability around the cut-off values. In patients fulfilling LS ≥ 14.5 kPa and PLT ≤ 110 × 10^9^/L, HRVs can be identified with more than 80% certainty, which was better than LS alone. More methods that may further improve the accuracy of the prediction of HRVs are needed in the future.

Factors affecting the accuracy of noninvasively diagnostic technique for HRVs are also issues of concern to clinical work [26, 27]. More than one-third of the patients in this study had a history of antiviral treatment. However, previous studies commonly excluded such patients from avoiding the interference of these factors [28, 29]. The problem is that patients with antiviral therapy history were left with limited ways to predict HRVs. Besides, the guidelines from the American Association for the study of liver diseases demonstrate that LS combining with platelet count to predict HRVs needs to be verified for cirrhotic patients who have achieved sustained virological response (SVR) [2]. An impressive result was that the history of antiviral therapy attenuated AUCs of 2D-SWE. Published data have proved that CSPH persists in most patients with hepatitis C virus-associated cirrhosis despite the SVR, indicating persistent risk of HRVs [30]. On the other hand, LS decreases rapidly after antiviral treatment, reflecting the remission of both hepatic inflammation and fibrosis [31]. Thus, the cut − off < 14.5 kPa performed poorly to rule out HRVs for patients with antiviral therapy history. However, we did not find a significant influence of antiviral treatment for predicting HRVs when combining LS with PLT. Moreover, a lower FNR was found for the combined algorithm than LS alone. This finding also validates the necessity of combining LS with PLT for predicting the presence of HRVs.

Our study has certain limitations. The size of the population is slightly smaller than those in previous studies. Moreover, except for excluding HRVs, we also want to find a method to rule in patients with a high risk of HRVs. Though higher ALT and TBIL levels may increase FPRs, however, it does not affect the most important characteristic of this algorithm, i.e., the safety profile. Since the level of ALT/AST of 90% (144/160) included patients were ≤2 × upper limit of normal, and 77.5% (124/160) of them were within the normal level of TBIL (<23.9 *μ*mol/L), we cannot further analyze the impact of these confounders. Future studies should expound their influence in the results. Additionally, the application of spleen stiffness (SS) has been attracted more and more attention [32], although it also has been reported that many factors will affect its values [33, 34]. However, this index also has the hope to be an accurate diagnostic indicator for HRVs [35] and may further improve the accuracy for predicting HRVs by LS alone [36]. More evidence is needed to compare the diagnostic efficacy using SS measured by different elastography techniques.

Last but not least, the practical implications of our findings can be concluded as follows. The most compelling evidence is that the LS value measured with 2D-SWE would safely avoid the need for additional endoscopy screening in patients with hepatitis B-related cirrhosis who are most likely to be free from HRVs. We recommend that the combination of LS with PLT is used for patients with antiviral history in predicting HRVs. Moreover, patients who meet the criteria LS ≥ 14.5 kPa and PLT ≤ 110 × 10^9^/L should be consent to perform endoscopic screening, and primary prophylaxis is required for patients with medium or large varices. The other patients in the “grey zone” need to consider combining other noninvasive tests or undergo endoscopy screening directly (Figure S2).

## Figures and Tables

**Table 1:** Characteristics of all patients included in the study.

Variables	Esophageal varices (*n* = 43)	No esophageal varices (*n* = 117)	*p* ^∗^	High-risk varices (*n* = 26)	No high-risk varices (*n* = 134)	*p* ^∗^
Age-yrs	52.70 ± 8.53	54.80 ± 9.96	0.221	52.38 ± 8.41	54.60 ± 9.82	0.284
Sex male-no.	40 (0.93)	94 (0.80)	0.054	24 (0.92)	110 (0.82)	0.196
Body mass index-kg/m^2^	24.45 ± 3.42	24.66 ± 3.38	0.721	24.05 ± 2.88	24.71 ± 3.47	0.354
ALT-IU/L	38.00 (28.00, 52.00)	31.00 (20.00, 50.00)	0.041	38.50 (27.75, 54.00)	32.00 (21.00, 50.00)	0.120
AST-IU/L	42.00 (30.00, 64.00)	33.00 (25.00, 44.00)	0.002	47.50 (38.25, 75.25)	34.00 (25.00, 44.25)	*p* < 0.001
TBIL-mg/dl	13.27 (10.50, 17.13)	8.58 (6.73, 11.88)	*p* < 0.001	14.74 (10.86, 18.63)	9.13 (6.89, 12.01)	*p* < 0.001
ALB-g/L	39.53 ± 6.19	43.30 (39.80, 45.75)	0.002	38.25 (32.90, 42.93)	43.20 (39.85, 45.73)	*p* < 0.001
PLT-×10^9^/L	113.77 ± 68.12	183.62 ± 63.27	*p* < 0.001	90.08 ± 52.94	179.35 ± 65.41	*p* < 0.001
GGT-IU/L	54.00 (31.00, 146.00)	45.00 (28.00, 83.50)	0.155	71.50 (30.00, 194.50)	46.00 (28.00, 82.25)	0.134
Prothrombin time -%	15.04 ± 1.85	14.07 ± 1.85	0.004	15.70 ± 1.96	14.06 ± 1.77	*p* < 0.001
INR	1.20 ± 0.18	1.10 ± 0.16	0.001	1.26 ± 0.20	1.10 ± 0.16	0.001
Serum creatinine-mg/dL	0.74 ± 0.15	0.76 ± 0.18	0.603	0.72 ± 0.16	0.76 ± 0.18	0.221
Child-Pugh class						
A-No.	34 (0.79)	112 (0.96)	*p* < 0.001	17 (0.65)	129 (0.96)	*p* < 0.001
B-No.	9 (0.21)	5 (0.04)		9 (0.35)	5 (0.04)	
MELD score	15.07 ± 3.15	12.88 ± 3.27	*p* < 0.001	15.68 ± 3.36	13.04 ± 3.21	*p* < 0.001
HBV DNA-log_10_IU/mL	3.19 (2.04, 4.44)	3.19 (1.90, 4.08)	0.400	2.97 (1.94, 4.63)	3.20 (1.94, 4.10)	0.682
Liver stiffness-kPa	14.60 (11.50, 20.30)	8.30 (7.10, 10.60)	*p* < 0.001	17.25 (14.38, 26.70)	8.85 (7.18, 11.43)	*p* < 0.001

Data are given as mean ± standard deviation or median (IQR) for continuous variables and percentage (%) for categorical variables. ^∗^Calculated from comparison between patients with and without esophageal varices or high-risk varices by the Student *t*-test, Mann–Whitney, or *χ*^2^ test as appropriate. Abbreviations: yrs: years; IU: international units; kPa: Kilopascal; No.: number.

**Table 2:** Diagnostic accuracy of 2D-SWE to discriminate all size varices and high-risk varices in patients with hepatitis B-related cirrhosis.

Patients with hepatitis B-related cirrhosis (*n* = 160)		
Diagnostic indexes	All size varices	High-risk varices
AUC (95% CI)	0.834 (0.767-0.888)	0.881 (0.820-0.927)
^‡^Cut-off values	11.4 kPa	14.50 kPa
Sensitivity	0.77 (0.61-0.88)	0.77 (0.56-0.91)
Specificity	0.80 (0.72-0.87)	0.91 (0.85-0.95)
PPV	0.59 (0.45-0.72)	0.63 (0.44-0.79)
NPV	0.90 (0.83-0.95)	0.95 (0.90-0.98)
Positive LR	3.9 (2.6-5.8)	8.6 (4.8-15.3)
Negative LR	0.3 (0.2-0.5)	0.3 (0.1-0.5)

Note: data in parentheses are 95% confidence intervals. ^‡^A cut-off was set to maximize the sum of sensitivity and specificity. Abbreviations: PPV: positive predictive value; NPV: negative predictive value; LR: likelihood ratio; CI: confidence interval.

**Table 3:** The combination of liver stiffness and platelet counts for ruling out and ruling in high-risk varices in patients with hepatitis B-related cirrhosis.

Patients with hepatitis B-related cirrhosis (*n* = 160)			
Noninvasive tests	^‡^LS < 14.5 kPa and PLT > 110 × 10^9^/L	^§^LS ≥ 14.5 kPa and PLT ≤ 110 × 10^9^/L	Combined rule in/out criteria
Sensitivity	0.96 (0.80-1.00)	0.54 (0.34-0.73)	0.93 (0.66-1.00)
Specificity	0.80 (0.72-0.86)	0.98 (0.93-0.99)	0.97 (0.92-0.99)
PPV	0.48 (0.34-0.62)	0.82 (0.56-0.95)	0.82 (0.56-0.95)
NPV	0.99 (0.95-1.00)	0.92 (0.85-0.95)	0.99 (0.95-1.00)
Positive LR	4.8 (3.4-6.7)	24.1 (7.4-77.8)	34.2 (11.1-105.3)
Negative LR	0.05 (0.01-0.3)	0.5 (0.3-0.7)	0.009 (0.001-0.07)
Spared endoscopies	108/160 (0.68)	17/160 (0.11)	125/160 (0.78)
HRVs missed	1/108 (0.01)	3/17 (0.18)	4/125 (0.03)

Note: data in parentheses are 95% confidence intervals. ^**‡**^Combined criteria for ruling out patients with HRVs. ^**§**^Combined criteria for ruling in patients with HRVs. Abbreviations: HRVs: high-risk varices; LS: liver stiffness; kPa: kilopascal; PPV: positive predictive value; NPV: negative predictive value; LR: likelihood ratio.

## Data Availability

Due to ethical restrictions imposed in the interest of participant confidentiality, data are available from the Ethics Committee of Shandong Provincial Hospital Affiliated to Shandong University for researchers who meet the criteria for access to confidential data (sdslyy@yeah.net).

## Abbreviations

LS: Liver stiffness
2D-SWE: Two-dimensional shear wave elastography
kPa: Kilopascals
EVs: Esophageal varices
HRVs: High-risk varices
ALT: Alanine aminotransferase
AST: Aspartate aminotransferase
GGT: Gamma glutamyltranspeptidase
TBIL: Total bilirubin
ALB: Albumin
PT: Prothrombin time
INR: International normalized ratio
PLT: Platelet count
MELD: Model for end-stage liver disease
PPV: Positive predictive value
NPV: Negative predictive value
LR: Likelihood ratio
AUC: Area under the curve
CI: Confidence interval
OR: Odds ratio
FPR: False-positive rate
FNR: False-negative rate.

## Supplementary Materials

Supplementary 1 Figure S1: nomograms to determine risk of high-risk varices based on liver stiffness and platelet count.

Supplementary 2 Figure S2: algorithm for high-risk varices screening in patients with hepatitis B-related cirrhosis.

Supplementary 3 Table S1: logistic stepwise regression analyses for variables associated with esophageal varices. Table S2: diagnostic efficacy of noninvasive indexes from logistic stepwise regression analyses.
