# Supplementary material for: The Combination of Shear Wave Elastography and Platelet Counts Can Effectively Predict High-Risk Varices in Patients with Hepatitis B-Related Cirrhosis
Source: Biomed Res Int. 2021 Apr 7;2021:6635963. doi: 10.1155/2021/6635963 (PMC8051526; doi:10.1155/2021/6635963)
Supplement: Supplementary 1 — Figure S1: nomograms to determine risk of high-risk varices based on liver stiffness and platelet count. [file 6635963.f1.docx]

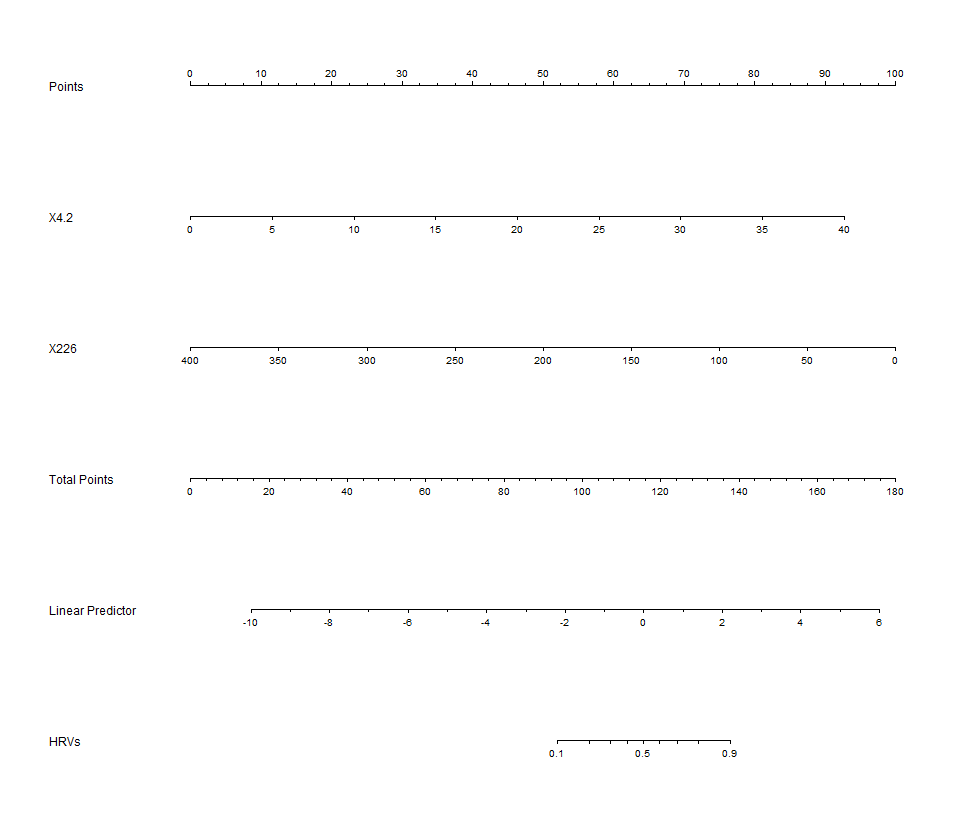


Figure S1 Nomograms to determine risk of high risk varices based on liver stiffness and platelet count
