# Supplementary material for: The Combination of Shear Wave Elastography and Platelet Counts Can Effectively Predict High-Risk Varices in Patients with Hepatitis B-Related Cirrhosis
Source: Biomed Res Int. 2021 Apr 7;2021:6635963. doi: 10.1155/2021/6635963 (PMC8051526; doi:10.1155/2021/6635963)
Supplement: Supplementary 2 — Figure S2: algorithm for high-risk varices screening in patients with hepatitis B-related cirrhosis. [file 6635963.f2.docx]

No endoscopy screening

Endoscopy

screening

Patients with hepatitis B related cirrhosis

LS values＜14.5 kPa

and PLT＞110 x10^9^/L

YES

NO

LS values≥14.5 kPa

and PLT≤110 x10^9^/L

NO

Combine with other noninvasive tests

YES

Figure S2 Algorithm for high risk varices screening in patients with hepatitis B related cirrhosis
