# Supplementary material for: The Combination of Shear Wave Elastography and Platelet Counts Can Effectively Predict High-Risk Varices in Patients with Hepatitis B-Related Cirrhosis
Source: Biomed Res Int. 2021 Apr 7;2021:6635963. doi: 10.1155/2021/6635963 (PMC8051526; doi:10.1155/2021/6635963)
Supplement: Supplementary 3 — Table S1: logistic stepwise regression analyses for variables associated with esophageal varices. Table S2: diagnostic efficacy of noninvasive indexes from logistic stepwise regression analyses. [file 6635963.f3.docx]

***Table S1*** Logisctic stepwise regression analyses for variables associated with esophageal varices^†^

|  | ALL size varices | | High-risk varices | |
| --- | --- | --- | --- | --- |
| Variables^‡^ | **OR(95% CI)** | **P** | **OR(95% CI)** | **P** |
| ALT-IU/L |  |  |  |  |
| AST-IU/L |  |  |  |  |
| TBIL-μmol/L |  |  |  |  |
| ALB-g/L |  |  | 0.810(0.684-0.960) | 0.015 |
| PLT-x10^9^/L | 0.988(0.980-0.996) | 0.004 | 0.981(0.968-0.994) | 0.005 |
| Prothrombin time-% |  |  |  |  |
| INR |  |  |  |  |
| Child-Pugh class |  |  |  |  |
| MELD Score | 1.208(1.042-1.402) | 0.013 | 1.538(1.140-2.074) | 0.005 |
| LS-kPa | 1.226(1.109-1.355) | P＜0.001 | 1.308(1.139-1.503) | P＜0.001 |

^†^All patients (n=160) were included in this analysis.

^‡^Variables in multivariate analysis only include noninvasive statistically significant (p＜0.1) indexes from Table 1.

Abbreviations: IU, international units; LS, liver stiffness; kPa, kiloPascal; OR, odds ratia; CI, confidence interval.

***Table S2*** Diagnostic efficacy of noninvasive indexes from logisctic stepwise regression analyses

| Patients with hepatitis B related cirrhosis (n=160) | | | | | | | | |
| --- | --- | --- | --- | --- | --- | --- | --- | --- |
| ALL size varices | | | | | | | | |
| Variable | **AUC(95%CI)** | **Cut-offs^‡^** | **Sensitivity** | **Specificity** | **PPV** | **NPV** | **LR+** | **LR-** |
| Liver stiffness | 0.834(0.767-0.888) | 11.4 | 0.77(0.61-0.88) | 0.80(0.72-0.87) | 0.59(0.45-0.72) | 0.90(0.83-0.95) | 3.9(2.6-5.8) | 0.3(0.2-0.5) |
| MELD score | 0.709(0.617-0.800) ^§^ | 14.71 | 0.60(0.44-0.75) | 0.80(0.72-0.87) | 0.53(0.38-0.68) | 0.85(0.77-0.91) | 3.1(2.0-4.8) | 0.5(0.3-0.7) |
| PLT | 0.795(0.704-0.887) | 112 | 0.65(0.49-0.79) | 0.92(0.86-0.96) | 0.76(0.59-0.88) | 0.89(0.81-0.93) | 8.5(4.4-16.5) | 0.4(0.3-0.6) |
| High-risk varices | | | | | | | | |
| Variable | **AUC(95%CI)** | **Cut-offs^‡^** | **Sensitivity** | **Specificity** | **PPV** | **NPV** | **LR+** | **LR-** |
| Liver stiffness | 0.881(0.820-0.927) | 14.50 | 0.77(0.56-0.91) | 0.91(0.85-0.95) | 0.63(0.44-0.79) | 0.95(0.90-0.98) | 8.6(4.8-15.3) | 0.3(0.1-0.5) |
| MELD score | 0.733(0.657-0.800) ^§^ | 14.57 | 0.65(0.44-0.83) | 0.73(0.65-0.80) | 0.32(0.20-0.46) | 0.92(0.85-0.96) | 2.4(1.6-3.6) | 0.5(0.3-0.8) |
| PLT | 0.868(0.806-0.917) | 112.00 | 0.77(0.56-0.91) | 0.87(0.81-0.92) | 0.54(0.37-0.71) | 0.95(0.90-0.98) | 6.1(3.7-9.9) | 0.3(0.1-0.5) |
| ALB | 0.746(0.671-0.811) ^§^ | 39.20 | 0.62(0.41-0.80) | 0.81(0.73-0.87) | 0.38(0.24-0.54) | 0.92(0.85-0.96) | 3.2(2.0-5.0) | 0.5(0.3-0.8) |

Note:Data in parentheses are 95% confidence intervals.

^‡^A cut-off was set to maximize the sum of sensitivity and specificity.

^§^P<0.05 for AUC comparison with liver stiffness.

Abbreviations: PPV, positive predictive value; NPV, negative predictive value; LR, likelihood ratio; CI, confidence interval.
